# How is masculinity related to body image? A cross-cultural investigation

**DOI:** 10.1186/2050-2974-1-S1-O48

**Published:** 2013-11-14

**Authors:** Debra Franko, Kristina Holmqvist Gattario, Ann Frisen, Matthew Fuller-Tyszkiewicz, Lina Ricciardelli, Zali Yager, Linda Smolak, Phillipa Diedrichs, Rachel F Rodgers, Heather Thompson-Brenner, Rebecca Shingleton

**Affiliations:** 1 Northeastern University, Boston, USA; 2 University of Gothenburg, Gothenburg, Sweden; 3 Deakin University, Melbourne, Australia; 4 La Trobe University, Victoria, Australia; 5 Kenyon University, OH, USA; 6 Centre for Appearance Research, University of the West of England, UK; 7 Boston University, MA, USA

## Introduction

Research indicates a relationship between masculinity and body image and highlights the importance of muscularity in young men's sense of masculinity. However, cross-cultural differences in these relationships have not been explored. We examined how conformity to masculine norms relates to attitudes toward muscularity, leanness, and thinness in men from Sweden, US, UK, and Australia and whether internalization of the muscular ideal mediated these relationships.

## Method

Over five hundred males [n = 142 (Sweden), 192 (US), 93 (UK), and 118 (Australia)] completed an online survey that included the Conformity to Masculine Norms Inventory, the Drive for Muscularity/Leanness/Thinness scales, and the Sociocultural Attitudes toward Appearance Questionnaire.

## Results

Path analyses confirmed that greater conformity to masculine norms predicted higher scores on measures of body change attitudes (drive for thinness, desire for leanness, and desire for muscularity), and identified internalization as a mediator of this relationship. However, structural invariance tests demonstrated significant cultural differences in the strength of these mediation effects. Further examination indicated that mediation effects among American, Australian, and Swedish males were comparable, whereas these effects were substantially weaker in the UK sample.

## Conclusions

Cultural differences in the role of internalization of the muscular ideal may inform research and prevention interventions.

This abstract was presented in the **Body Image** stream of the 2013 ANZAED Conference.

